# Editorial: Precision oncology in the era of CRISPR-Cas9 technology

**DOI:** 10.3389/fgene.2024.1506627

**Published:** 2024-10-18

**Authors:** Athar Khalil

**Affiliations:** Department of Genetics and Genome Sciences, Case Western Reserve University, Cleveland, OH, United States

**Keywords:** Crisper Cas9, PROTACs, DNA editing systems, cancer treatment, precision oncology

Cancer remains a leading cause of morbidity and mortality worldwide, posing one of the greatest challenges in modern medicine. The rapid evolution of precision oncology has ushered in a new era of hope for patients, targeting specific molecular mechanisms of cancer while minimizing collateral damage to healthy cells. At the heart of this revolution is CRISPR-Cas9 technology, a tool with extraordinary precision in editing DNA, which has fundamentally reshaped our approach to understanding and treating cancer. Alongside other emerging technologies, such as PROTACs (PROteolysis Targeting Chimeras) and AI-driven systems, CRISPR promises to usher in a new phase of personalized cancer therapy. In this editorial, we explore the pivotal role of CRISPR in cancer understanding and discuss the synergies that could further elevate precision oncology.

CRISPR-Cas9 has earned widespread acclaim for its ability to efficiently and accurately modify DNA sequences. Since its discovery, this genome-editing tool has transformed research and clinical applications in oncology by enabling scientists to directly target and disrupt cancer-causing genes. Preclinical studies have demonstrated its utility in targeting oncogenes, tumor suppressor genes, and immune checkpoint molecules—cornerstones of cancer biology (Sharma and Giri). By modifying or knocking out specific genes, CRISPR has already shown promise in boosting the efficacy of adoptive cell therapies like CAR-T cells, where editing inhibitory genes can enhance immune responses (Ravichandran and Maddalo).

However, the potential applications of CRISPR-Cas9 extend far beyond gene editing in immune cells. The technology is being used to modify the tumor microenvironment, regulate gene expression in real-time, and identify drug resistance mechanisms (Li et al.; Sharma and Giri). In cancer immunotherapy, where immune checkpoint inhibitors (ICIs) have achieved remarkable success, CRISPR-based screening approaches are uncovering new targets that could amplify the efficacy of these therapies. For instance, *in vivo* CRISPR screens have revealed novel gene networks that regulate immune responses to tumors, opening the door for new combination therapies. Additionally, CRISPR activation and interference libraries (CRISPRa and CRISPRi) have helped map the genetic underpinnings of cancer resistance to natural killer (NK) and T cell-mediated cytotoxicity, highlighting new avenues for therapeutic intervention. As these CRISPR techniques continue to evolve, they promise to expand the scope of immunotherapy in ways previously unimaginable (Wang et al.).

While CRISPR’s ability to target oncogenic mutations with precision is unparalleled, it is not without challenges. Off-target effects, where unintended sections of the genome are edited, remain a significant concern, particularly in long-term therapies. Chromosomal rearrangements and immune reactions to CRISPR components pose additional hurdles. Despite these concerns, emerging techniques such as base editing and prime editing offer more refined methods for precise DNA modifications (Ravichandran and Maddalo). These advancements are already showing potential for reducing off-target effects and enhancing the specificity of gene edits, thus increasing the safety and efficacy of CRISPR-based therapies in clinical settings.

Beyond therapeutic applications, CRISPR is making inroads into cancer diagnostics. The development of CRISPR-based platforms for detecting cancer-specific mutations in blood samples is revolutionizing liquid biopsy approaches. Technologies like SHERLOCK and DETECTR, which use CRISPR-associated proteins to detect DNA or RNA mutations, are providing non-invasive, highly sensitive methods for early cancer detection. These tools are poised to become key assets in the fight against cancer, allowing for earlier diagnosis and more personalized treatment strategies (Ravichandran and Maddalo).

Parallel to the rise of CRISPR is the advent of PROTACs, another technology with transformative potential in precision oncology. Whereas CRISPR modifies DNA, PROTACs target proteins for degradation via the ubiquitin-proteasome system, enabling the selective elimination of oncogenic proteins. By degrading proteins that are traditionally considered “undruggable,” this technology have opened up new possibilities for targeting cancer drivers that were previously out of reach. The ability to administer PROTACs in low oral doses and their high specificity for target proteins make them an attractive complement to CRISPR-based therapies. Rather than competing technologies, CRISPR and PROTACs offer synergistic advantages: CRISPR addresses genetic aberrations, while the latter remove the resulting protein products, creating a comprehensive strategy for tackling cancer on multiple fronts (Kanbar et al.).

The integration of artificial intelligence (AI) into these approaches further amplifies their potential. AI-driven algorithms are already being used to optimize CRISPR and PROTAC designs, predicting on-target and off-target effects with increasing accuracy. Machine learning models are assisting in refining CRISPR guide RNAs, reducing the risk of unintended gene edits, while also improving the design of PROTAC molecules for better drug permeability and efficacy. AI’s role in precision oncology extends beyond computational design—it is accelerating drug discovery, helping identify new therapeutic targets, and enabling more efficient high-throughput screening. By combining AI with DNA and protein editing technologies, the pace of innovation in cancer treatment is set to quicken, providing patients with more personalized and effective treatment options (Kanbar et al.).

Despite these advances, significant barriers remain to the full clinical translation of CRISPR and PROTAC technologies. Ethical concerns surrounding gene editing, particularly in relation to germline modifications, continue to provoke debate. Additionally, Research Topic such as delivery mechanisms for CRISPR components, potential immune reactions, and scalability of PROTAC synthesis must be addressed before these technologies can become mainstream clinical tools. Encouragingly, progress is being made on several fronts. Nanoparticle-based delivery systems are showing promise for enhancing the precision of CRISPR-based therapies, while innovations in on-chip screening platforms are facilitating faster, more efficient drug development (Kanbar et al.).

In conclusion, CRISPR-Cas9 has fundamentally transformed the landscape of precision oncology, offering unprecedented possibilities for targeting cancer at the genetic level. When combined with complementary technologies like PROTACs and AI-driven innovations, CRISPR holds the potential to unlock new frontiers in personalized cancer therapy. The future of cancer treatment lies not in a single technology but in the integration of multiple, synergistic approaches that address the complexity of cancer biology. As we continue to refine these tools and overcome the challenges of clinical translation, the era of truly personalized, targeted cancer treatments is closer than ever. The next decade will be critical in determining how these technologies shape the future of oncology, with the promise of more effective, efficient, and personalized interventions that can dramatically improve patient outcomes.

